# Presentation and mortality of patients hospitalised with acute heart failure in Botswana

**DOI:** 10.5830/CVJA-2016-067

**Published:** 2017

**Authors:** Julius Chacha Mwita, Bernard Omech, Koketso Lister Majuta, Marea Gaenamong, Tommy Baboloki Palai, Mosepele Mosepele, Matthew J Dewhurst, Mgaywa GMD Magafu, Julius Chacha Mwita, Monkgogi Goepamang, Bernard Omech, Tommy Baboloki Palai, Mosepele Mosepele, Yohana Mashalla

**Affiliations:** Department of Internal Medicine, University of Botswana, Gaborone, Botswana; Department of Internal Medicine, University of Botswana, Gaborone, Botswana; Department of Internal Medicine, University of Botswana, Gaborone, Botswana; Department of Internal Medicine, University of Botswana, Gaborone, Botswana; Department of Internal Medicine, University of Botswana, Gaborone, Botswana; Department of Internal Medicine, University of Botswana, Gaborone, Botswana; Department of Cardiology, North Tees and Hartlepool NHS Foundation Trust, UK; Department of Family Medicine and Public Health, University of Botswana, Gaborone, Botswana; Department of Internal medicine, Princess Marina Hospital, Gaborone,Botswana; Department of Internal medicine, Princess Marina Hospital, Gaborone,Botswana; Department of Internal medicine, Princess Marina Hospital, Gaborone,Botswana; Department of Internal medicine, Princess Marina Hospital, Gaborone,Botswana; Department of Internal medicine, Princess Marina Hospital, Gaborone,Botswana; Department of Biomedical Sciences, University of Botswana, Gaborone, Botswana

**Keywords:** acute heart failure,, in-hospital mortality,, length of hospital stay,, outcomes,, Botswana

## Abstract

**Introduction:**

Heart failure is a common cause of hospitalisation and therefore contributes to in-hospital outcomes such as mortality. In this study we describe patient characteristics and outcomes of acute heart failure (AHF) in Botswana.

**Methods:**

Socio-demographic, clinical and laboratory data were collected from 193 consecutive patients admitted with AHF at Princess Marina Hospital in Gaborone between February 2014 and February 2015. The length of hospital stay and 30-, 90- and 180-day in-hospital mortality rates were assessed.

**Results:**

The mean age was 54 ± 17.1 years, and 53.9% of the patients were male. All patients were symptomatic (77.5% in NYHA functional class III or IV) and the majority (64.8%) presented with significant left ventricular dysfunction. The most common concomitant medical conditions were hypertension (54.9%), human immuno-deficiency virus (HIV) (33.9%), anaemia (23.3%) and prior diabetes mellitus (15.5%). Moderate to severe renal dysfunction was detected in 60 (31.1%) patients. Peripartum cardiomyopathy was one of the important causes of heart failure in female patients. The most commonly used treatment included furosemide (86%), beta-blockers (72.1%), angiotensin converting enzyme inhibitors (67.4%), spironolactone (59.9%), digoxin (22.1%), angiotensin receptor blockers (5.8%), nitrates (4.7%) and hydralazine (1.7%). The median length of stay was nine days, and the in-hospital mortality rate was 10.9%. Thirty-, 90- and 180-day case fatality rates were 14.7, 25.8 and 30.8%, respectively. Mortality at 180 days was significantly associated with increasing age, lower haemoglobin level, lower glomerular filtration rate, hyponatraemia, higher N-terminal pro-brain natriuretic peptide levels, and prolonged hospital stay.

**Conclusions:**

AHF is a major public health problem in Botswana, with high in-hospital and post-discharge mortality rates and prolonged hospital stays. Late and symptomatic presentation is common, and the most common aetiologies are preventable and/or treatable co-morbidities, including hypertension, diabetes mellitus, renal failure and HIV.

## Introduction

The prevalence of heart failure (HF) is increasing in Africa, adding to the already existing burden of infectious diseases and making HF a common cause of hospitalisation on the continent.^1^,^2^ HF is one of the primary reasons for regular hospital visits and admissions, accounting for about three to 7% of admissions in Africa.^3^ In spite of advances in treatment, patients admitted with acute heart failure (AHF) have outcomes that are worse than many types of cancer.^4^,^5^ In Africa, where the majority of patients are likely to present late and with severe symptoms, the in-hospital mortality rate of AHF ranges from nine to 12.5%, which is considerably higher than in developed countries.^6^ Even after hospital discharge, case fatality rates for HF remain high, with mortality rates of more than 25, 40 and 75% at three months, one year and five years after diagnosis, respectively.^7^-^9^

Although HF management has advanced in the Western world, in many developing countries, including Botswana, the benefits may not be evident for several reasons, including insufficient human resources, lack of appropriate medications and discontinuity of care. This study aimed to describe clinical profiles and outcomes in patients with AHF admitted at Princess Marina Hospital (PMH) in Gaborone, Botswana.

## Methods

This was an observational study conducted at PMH, Botswana’s major tertiary and referral hospital, with a catchment population of 231 592 in Gaborone, plus patients referred from other areas of the country.^10^ The study was granted ethical clearance by the University of Botswana and PMH institutional review boards, and permission to carry out the study was obtained from the Ministry of Health. Written informed consent was obtained before data collection from all participants, or their relatives, in cases where the patient was unable to consent.

Consecutive AHF patients aged 18 years of age or older admitted to the hospital between February 2014 and February 2015 were enrolled in the study. HF was defined according to the criteria of the European Society of Cardiology (ESC), and both decompensated HF in patients with a previous HF diagnosis and new-onset AHF were included.^11^ Patients were excluded if they had other diseases with a short-term prognosis, such as malignancy or World Health Organisation stage 4 HIV infection.

From the enrolled patients, symptoms and signs of HF were ascertained, and the admission functional status was assessed using the New York Heart Association (NYHA) classification.12 Any pre-hospital medical history of atrial fibrillation, valvular heart disease, diabetes mellitus, hypertension, HIV infection and cerebrovascular disease was also recorded.

On the day of enrolment, three blood pressure measurements were obtained and averaged.^13^ The blood pressure measurement made on admission was also recorded. A patient was considered hypertensive on the basis of a self-reported history of hypertension and/or the use of blood pressure-lowering medications or a sustained blood pressure ≥ 140/90 mmHg during the course of the admission.^14^

Complete blood counts, serum electrolytes, urea, creatinine, uric acid and N-terminal pro-brain natriuretic peptide (NT-proBNP) analyses were performed on all enrolled patients. Moderate to severe renal failure was diagnosed by an estimated glomerular filtration rate (eGFR) of less than 60 ml/min/1.73 m^2^ at admission and/or by patients being on dialysis.^1^ Patients whose haemoglobin values were less than 10 g/dl were classified as having anaemia.^7^,^1^ Testing for HIV was done for patients whose sero-status was unknown.

Echocardiography using a Vivid S™ S6 machine (GE Healthcare view, USA) was performed on all patients by two cardiologists (JM and MG) according to the American Society of Echocardiography guidelines.^16^ Two-dimensional M-mode measurements of left ventricular (LV) internal dimension, interventricular septal thickness and posterior wall thickness were made at end-diastole and end-systole.^16^,^17^ M-mode measurements for the left atrial diameters were obtained at end-systole.

Left ventricular ejection fraction (LVEF) was calculated from left ventricular volumes obtained at end-diastole and end-systole using the modified biplane Simpson’s rule in the apical four- and two-chamber views.^16^ LVEF < 45% was used to define significant LV systolic dysfunction, whereas patients with LVEF > 45% were assessed as having HF with preserved ejection fraction. Right ventricular dysfunction was assessed by a tricuspid annular plane systolic excursion (TAPSE) < 16 cm.^17^

Available clinical and echocardiographic data were used to assign a likely primary aetiology to each patient based on the ESC guidelines and the Heart of Soweto study definitions.^11^,^18^ Ischaemic HF was determined by the presence of LV systolic dysfunction, regional wall-motion abnormality, electrocardiographic abnormalities, and angiographically confirmed diagnosis of coronary artery disease.^18^ Patients with LV systolic dysfunction and dilated left ventricle (LVEDD > 55 mm) of indeterminate cause were classified as having idiopathic dilated cardiomyopathy.^18^

Peripartum cardiomyopathy was diagnosed in patients with echocardiographic features of dilated cardiomyopathy without a demonstrable cause, and if disease presented for the first time within the last trimester of pregnancy or in the first five months postpartum.^19^ Other causes of HF included pericardial disease, congenital heart disease, amyloidosis, hypertrophic cardiomyopathy, restrictive cardiomyopathy, thyroid heart disease and HIV infection.^20^

Hospital length of stay (LOS) and in-hospital mortality were assessed for each participant. LOS was defined as the number of days from hospital admission to discharge. After discharge, patients were followed up at the PMH out-patient cardiac clinic for clinical evaluation and medication adjustment or titration.

HF medications at discharge or at end-of-study follow up were documented, and patients were contacted whenever they missed their scheduled out-patient appointments, to reschedule for another appointment. In the event that participants were not contactable, the next of kin/nominated contacts were contacted. For participants who relocated to other health facilities, their information was retrieved from the nationwide electronic medical records (EMR) database, which contains clinical notes, laboratory results, pharmacy data and information on dates of patient clinic and hospital visits.

Information on mortality was collected at 30, 90 and 180 days by telephone contact with their next of kin/nominated contacts and/or from the EMR. Participants who could not be contacted or traced through the EMR after discharge were declared as lost to follow up. Other patients were censored at the last available contact or clinic visit.

## Statistical analysis

All data were analysed using SPSS version 23.0 for Windows (SPSS Inc, Chicago, IL, USA), and summary statistics were calculated for all patient variables. Continuous variables are presented as means ± one standard deviation (SD) or medians. For non-continuous variables, absolute and relative frequencies (%) were used. Comparisons between normally distributed continuous variables were performed using the Student’s t-test or Kruskal–Wallis test. Associations between categorical variables were tested with contingency tables and Pearson’s chi-squared test; p-values less than 0.05 were considered statistically significant.

## Results

From the 202 patients admitted with HF during the study period, 193 (95.5%) were enrolled. Nine patients were excluded from the study because they failed to meet the inclusion criteria, four were unwilling to participate, two died and one was transferred to another hospital before enrolment. More than half of the enrolled patients (56%) were referrals from health facilities outside Gaborone.

Table 1 shows the clinical and demographic characteristics of the study population. Most patients were black Africans (98.4%). Cigarette and alcohol use was found in 13 and 15%, respectively. The mean age of the patients [± standard deviation (SD)] was 54.2 ± 17.1 years, ranging from 20 to 89 years. Smoking was significantly more common in the men (18.3%) than in women (6.7%). All patients were symptomatic, and the majority (77.5%) presented with dyspnoea (NYHA functional class III or IV), peripheral oedema, orthopnoea, palpitations and basal crepitations. Overall, the median (IQR) systolic and diastolic blood pressures were 120.0 (103.8–133.5) and 74 (67–81.5) mmHg, respectively. Because of cost limitations, the NT-proBNP level was determined in only 107 patients, with a median value (IQR) of 3 314 (1 360–6 506) pg/ml.

**Table 1:** Clinical and demographic characteristics of patients admitted with heart failure

Characteristics	n = 193
Mean age (years) (SD)	54.2 ± 17.1
Male gender, n (%)	104 (53.9)
Medical history, n (%)	
Hypertension	106 (54.9)
Prior type 2 diabetes	30 (15.5)
Renal failure	28 (15.0)
Rheumatic heart disease	21 (10.9)
Ischaemic heart disease	11 (5.7)
Stroke	19 (9.8)
Atrial fibrillation	19 (9.8)
HIV positive	61 (33.9)
Symptoms, n (%)	
Shortness of breath	178 (92.2)
NYHA II	40 (22.5)
NYHA III	94 (52.8)
NYHA IV	44 (24.7)
Orthopnoea	151 (78.2)
Peripheral oedema	148 (76.7)
Paroxysmal nocturnal dyspnoea	152 (78.8)
Physical findings	
Mean heart rate (bpm) (SD)	95.1 ± 21.2
Median systolic blood pressure (mmHg) (Q1–Q3)	120.0 (103.8–133.5)
Median diastolic blood pressure (mmHg) (Q1–Q3)	74 (67–81.5)
Murmur, n (%)	76 (39.4)
Cyanosis, n (%)	7 (3.6)
Pedal oedema, n (%)	132 (68.4)
Elevated jugular venous pressure, n (%)	127 (65.8)
S3 gallop, n (%)	72 (37.3)
Basal crepitation, n (%)	126 (65.3)
Hepatomegaly, n (%)	100 (52.6)
Ascites, n (%)	43 (23.8)
Pleural effusion, n (%)	31 (16.1)
Laboratory tests	
Mean haemoglobin (g/dl) (SD)	12.0 ± 2.96
Creatinine (μmol/l) median (Q1–Q3)	98.0 (70–137.5)
Urea (mmol/l) median (Q1–Q3)	8.3 (4.9–13.7)
Mean sodium (mmol/l) (SD)	134.1 ± 6.8
Mean potassium (mmol/l) (SD)	4.4 ± 0.9
eGFR (ml/min/1.73 m2) median (Q1–Q3)	75.9 (52.5–112.4)
Echocardiography	
Mean LVEF (%) (SD)	41.8 ± 20.0
Mean LA (mm) (SD)	43 ± 9
Mean IVSD (mm) (SD)	12.9 ± 4.1

LVEF, left ventricular ejection fraction; eGFR, glomerular filtration rate; IQR, interquartile range; SD, standard deviation; Q, quartile; NYHA, New York Heart Association functional class; LVEF, left ventricular ejection fraction, LA, left atrium; IVSD, interventricular septum diameter.

More than one-half (54.9%) of the patients were hypertensive. Hypertension was more commonly reported in the women than the men (64.0 vs 47.1%, p < 0.05). A prior diagnosis of diabetes mellitus was present in 30 (15.5%) patients and often coexisted with hypertension. Moderate to severe renal dysfunction was detected in 60 (31.1%) patients. Forty-five (23.3%) patients had a haemoglobin level < 10 g/dl, a finding that was commonly seen among those with chronic kidney disease. Overall, anaemia was more common in the women than men. HIV results were available for 180 (93.3%), and about a third (33.9%) of these patients were HIV positive.

The mean LVEF was 41.7%, and about two-thirds (64.8%) of the patients had HF with significant systolic dysfunction (LVEF < 45%). Seventy-three (37.8%) patients presented with severely depressed LV function (LVEF < 30%), which was more common in men than women (44.2 vs 30.3%, p < 0.05). Left atrial diameter was enlarged to a mean value of 43 mm, and moderate to severe mitral and tricuspid regurgitation was common (31.6 and 40.4%, respectively). Other less common valvular disorders found were mitral stenosis, aortic regurgitation and aortic stenosis. The commonest causes of HF were hypertensive heart disease (40.4%), dilated cardiomyopathy (19.6%), cor pulmonale (9.8%), valvular heart disease (9.3%) and pericardial disease (6.2%).

Overall, right ventricular dysfunction was common, present in 86 (44.6%) patients, and often coexisted with left ventricular systolic dysfunction. Pericardial disease, dilated cardiomyopathy and right HF were more common in men while hypertensive and valvular HF were more common in women. Although peripartum cardiomyopathy only accounted for 4.1% of all cases of HF, it turned out to be the third commonest cause of HF among female patients, occurring in eight (9%) of all female patients. The mean age of patients with peripartum cardiomyopathy was significantly lower than the other female patients (32 ± 7.3 vs 57 ± 16.8 years, p < 0.01). Patients with pericardial disease were more likely to be HIV positive than those with other types of HF. Ischaemic heart disease was found in 5.7% of the patients and was more common in women and patients with hypertension and diabetes. Other causes of HF in the cohort included amyloidosis, hypertrophic cardiomyopathy, thyroid heart disease and congenital heart disease.

Table 2 shows patient outcomes in the wards, discharge medications, and outcomes over the six months of postdischarge follow up. Overall, diuretics, beta-blockers, angiotensin converting enzyme (ACE) inhibitors or angiotensin receptor blockers, and spironolactone were often prescribed to patients at discharge. The most commonly used medications were carvedilol, bisprolol, enalapril, telmisartan and furosemide.

**Table 2:** Outcomes and discharge medications of patients admitted with acute heart failure at Princess Marina Hospital

Discharge medication	Number (%)
Diuretics	148 (86)
Beta-blockers	124 (72.1)
ACE inhibitors	116 (67.40)
Angiotensin receptor blockers	10 (5.8)
Spironolactone	103 (59.9)
Digoxin	38 (22.1)
Nitrate	8 (4.7)
Hydralazine	3 (1.7)
Outcome	
Median length of hospital stay (IQR)	9 (5–15)
In-hospital mortality (n = 193)	21 (10.9)
30-day mortality (n = 190)	28 (14.7)
90-day mortality (n = 182)	47 (25.8)
180-day mortality (n = 181)	56 (30.9)

In-hospital mortality rate of HF patients was 10.9%, and was associated with hyponatraemia (p = 0.023), elevated NT-proBNP (p = 0.001) and urea levels (p = 0.013), and hyperuricaemia (p = 0.036). The median length of stay was nine days (IQR 5–15). The LOS was similar regardless of the NYHA functional status of HF after hospital discharge. After 30 days, three (1.6%) patients could not be traced telephonically and were declared lost to follow up.

Seven patients (4.1%) died within 30 days of discharge from hospital. Overall, 28/190 (14.7%) patients died within 30 days of admission. By the 90th day after admission, 10 (5.2%) patients were lost to follow up, and of the rest, 47/183 (25.7%) were deceased. A total of 11 (5.7%) were lost to follow up by six months of admission, and the 180-day case fatalities from HF were 30.8%. Mortality at 180 days was significantly associated with increasing age, lower haemoglobin level, lower eGFR, hyponatraemia, higher NT-proBNP levels, and prolonged hospital stay (Table 3).

**Table 3:** Associations between demographic and medical characteristics of the patients and mortality outcome at the end of the follow-up period (six months post-admission)

	Outcome		
Characteristic	Died (n = 56)	Survived (n = 125)	p-value
Age (years) mean (SD)	59.8 (16.5)	51.93 (16.547)	0.004†
Male gender, n (%)	32 (57.1)	65 (52)	0.60*
Medical history, n (%)			
Hypertension	27 (48.2)	72 (57.6)	0.200*
Diabetes	7 (12.5)	21 (16.8)	0.515*
Rheumatic heart disease	4 (7.1)	10 (7.9)	1.00*
Ischaemic heart disease	3 (5.4)	15 (12)	0.281*
Stroke/TIA	4 (7.1)	13 (10.4)	0.591*
Atrial fibrillation	7 (12.5)	11 (8.8)	0.431*
HIV positive	16 (28.6)	41 (32.8)	0.306*
Clinical history			
Median SAP (mmHg)	120.5 (108–131.4)	120.0 (101.5–141.3)	0.887‡
Mean DAP (mmHg)	75.3 (67–79.9)	74 (66.0– 85.3)	0.878‡
LVEF (%)	41.9 ± 20.7	41.8 ± 20.2	0.975†
Haemoglobin (g/dl)	11.2 ± 3.1	12.4 ± 2.7	0.010†
MCV (%)	87.9 ± 9.5	89.3 ± 10.9	0.337†
eGFR (ml/min/1.73 m2), median (IQR)	70.95 (41.7–95.6)	84.8 (55.9–113.6)	0.043‡
Sodium (mEq/l)	132.0 ± 8.0	135.1 ± 6.1	0.010†
LOS (days), median (IQR)	11 (6.0–19.8 )	7 (5–12.3)	0.005‡
Creatinine (μmol/l), median (IQR)	116.5 (80.5–149.0)	96.0 (66.8–130.8)	0.041‡
Urea (mmol/l), median (IQR)	11.4 (6.6–18.9)	7.1 (4.7–12.0)	0.002‡
NT-proBNP (pg/ml), median (IQR)	6597 (4340.0–18810.3)	2739.0 (998.5–4656.0)	< 0.001‡

*Chi- squared; ‡Kruskal–Wallis, ^†^Student’s t-test.

TIA, transient ischaemic attack; SAP, systolic arterial pressure; DAP, diastolic arterial pressure; LVEF, left ventricular ejection fraction; MCV, mean corpuscular volume; eGFR, estimated glomerular filtration rate; LOS, length of stay; NT-proBNP, N-terminal pro-brain natriuretic peptide.

## Discussion

This is the first observational study of acute HF in Botswana, and it confirms the findings of previous studies that are unique to the African setting. Contrary to the situation in developed countries, where HF patients present at a much older age, with most cases recorded around the seventh and eighth decades of life, our cohort comprised relatively young patients (mean age 54 years).^21^ Similar to previous studies of AHF patients in Africa, our data have shown that HF affects young and middle-aged Africans in their most productive period of life.^7^,^18^,^22^-^25^

The young age at presentation is not surprising because of the predominance of non-ischaemic causes of HF among black Africans, such as hypertension, which often occur early in life and remain undetected, or if detected are inadequately treated. In our cohort, ischaemic HF was found in only 5.7% of the patients. As expected, a significant proportion of our patients was symptomatic, with left ventricular systolic dysfunction, and was predominantly men.^7^7,22,23 More than three-quarters (77.5%) presented in NYHA functional class III or IV, a finding which is in agreement with previous reports.^6^

One of the most striking features of this cohort was the relatively high prevalence of HIV-seropositive patients. The prevalence of HIV seropositivity (33.9%) reported in this study is far higher than in the previous largest HF study among HIV-poitive patients in South Africa.^20^ The observed high prevalence of HIV in our cohort may partly be explained by a relatively higher prevalence of HIV in Botswana than in South Africa.^26^ Botswana has the world’s third-highest HIV infection rate in the world after Swaziland and Lesotho, with an adult prevalence rate of about 24.4% in 2012.^26^

This high prevalence of HIV infection in our cohort provides additional evidence of the confluence of non-communicable and infectious diseases as co-morbidities among HF patients in Africa. HIV-associated cardiomyopathy, pulmonary hypertension, pericardial disease and accelerated atherosclerosis are known to occur frequently in HIV-positive patients.^27^ Our data reaffirmthat hypertension, diabetes, renal failure and anaemia are still common and contribute significantly to the aetiological burden of HF in Africa. These co-morbidities are not only the likely aetiologies of HF, but are also factors that affect the clinical course of the disease, and should be concurrently addressed at the time of admission.^28^

Consistent with previous reports from sub-Saharan Africa, hypertensive heart disease, dilated cardiomyopathy, cor pulmonale (right heart disease), peripartum cardiomyopathy, pericardial disease and valvular heart disease were the common causes of HF in our cohort.^6^,^7^,^18^,^20^,^22^,^23^,^25^,^27^ Ischaemic HF, however, was uncommon in our cohort, and patients with pericardial disease were more likely to be HIV positive than those with other types of HF

In our study, peripartum cardiomyopathy was the third commonest aetiology of HF among females. Although the mean age of patients with peripartum cardiomyopathy was similar to previous studies in Africa, the reported prevalence (9%) was lower than that reported in other African studies, where 13 to 60% of admissions for HF in females were related to peripartum cardiomyopathy.^25^,^29^,^30^ Nevertheless, consistent with the above studies, it is clear that peripartum cardiomyopathy is one of the important causes of HF among female patients in Africa.

The present study demonstrates that patients with HF are at risk for adverse clinical outcomes, which ranks HF among the major causes of death of cardiovascular origin in Africa.6 The in-hospital mortality rate (10.9%) in our cohort is similar to the nine to 12.5% case fatality rates reported elsewhere in Africa, but was higher than reports from high-income countries.^6^,^21^,^24^, ^31^

Generally, African-Americans have been reported to have a lower in-hospital mortality rate compared to Caucasians, and the same results could be expected among HF patients in Africa if the quality of care was similar.^31^ However, in resourcepoor settings such as ours, there is inconsistent availability of HF medications, limited access to intensive care services, and non-availability of advanced HF treatments such as cardiac resynchronisation therapy and assist devices, which are commonly used in the developed world. The high mortality rates in the current study and other African studies should therefore be taken as a call for improvement in the care of patients with HF in sub-Saharan Africa.

The LOS is another measure of quality of in-hospital care of HF and is directly related to cost.32 The median LOS of nine days in our cohort is similar to previous studies in Africa and Europe, but more than twice that reported in North America.^7^,^21^,^24^,^31^ The LOS found in our study and probably other African studies may be explained by multiple factors that include a significant coexistence of other acute or chronic medical conditions, occasional interruption of treatment due to unavailability of medication at hospitals, non-compliance, low rates of intensive care admission, and patients’ non-medical problems requiring intervention. Being a tertiary hospital in Botswana, PMH receives patients with severe forms of HF, including patients referred from distant hospitals, whose hospital stays may be prolonged by lack of timely transport back to their referring hospitals or residence.

After discharge, case fatality rates among those with HF are reported to be high, with up to 40% of those with severe HF dying within one year.^7^,^8^ In our study, about a third (30.9%) of patients died within six months of admission, a rate that is significantly higher than the 180-day mortality rate of 17.8% reported in the large THESUS-HF study.^5^ The difference in mortality rates may partly be explained by the fact that the THESUS-HF study was performed in different settings with variable patient presentations and mortality rates.^7^

Several co-morbidities that were prevalent among our patients, and are known to independently increase the risk of mortality among HF patients were hypertension, diabetes, renal failure and anaemia.^28^ Overall, our study showed that in-hospital and post-discharge mortality rates were higher in patients who had longer lengths of hospital stay, hyponatraemia, older age, lower haemoglobin level, higher NT-proBNP level, and lower eGFR. These poor prognostic factors have also been reported in other studies.^28^

This study was undertaken in a small town and hence is limited by the relatively small number of patients. However it provides useful findings, opening new avenues for future studies on HF. Because our cohort was selected from a tertiary hospital, it is likely to over-represent those with severe HF. For various reasons, we could not follow up all our patients at our clinic after discharge, and it was not possible to gather information on their treatment. Therefore, the influence of the differences in out-patient care on patients’ outcomes could not be assessed. All deaths were assumed to be attributable to HF, which is also likely to be an overestimation because of other significant medical co-morbidities that were prevalent in our patients.

## Conclusion

This study has demonstrated high morbidity and mortality rates among patients admitted to a tertiary hospital in Botswana for AHF. Both non-communicable (hypertension) and infectious diseases (HIV) are common among HF patients and often coexist. As mortality rates among HF patient remain high after admission, efforts should be made to improve HF management, both on an in-patient basis and in the community following discharge, in order to help improve prognosis.
